# Rapid Identification of Clinically Relevant *Candida* spp. by I-dOne Software Using Attenuated Total Reflectance Fourier Transform Infrared (ATR-FTIR) Spectroscopy

**DOI:** 10.3390/jof11010040

**Published:** 2025-01-07

**Authors:** Iacopo Franconi, Roberta Fais, Cesira Giordano, Benedetta Tuvo, Chiaramaria Stani, Arianna Tavanti, Simona Barnini, Antonella Lupetti

**Affiliations:** 1 Department of Translational Research and New Technologies in Medicine and Surgery, University of Pisa, Via San Zeno 37-39, 56127 Pisa, Italy; iacopo.franconi@phd.unipi.it (I.F.); roberta.fais@uslnordovest.toscana.it (R.F.); b.tuvo@studenti.unipi.it (B.T.); 2 SD Microbiology Bacteriology, Azienda Ospedaliero-Universitaria Pisana, 56126 Pisa, Italy; cesira.giordano@ao-pisa.toscana.it (C.G.); s.barnini@ao-pisa.toscana.it (S.B.); 3 Elettra Sincrotrone Trieste S.C.p.A., 34149 Trieste, Italy; chiaramaria.stani@ceric-eric.eu; 4 Department of Biology, University of Pisa, 56127 Pisa, Italy; arianna.tavanti@unipi.it

**Keywords:** ATR-FTIR spectroscopy, *Candida* spp., I-dOne software, *Candida* spp. identification

## Abstract

Attenuated total reflectance Fourier transform infrared (ATR-FTIR) spectroscopy is a spectrum-based technique that quantifies the absorption of infrared light by molecules present in the microbial cell. The aim of the present study was to evaluate the performance of the ATR-FTIR spectroscopic technique via I-dOne software (Version 2.0) compared with the MALDI-TOF MS in identifying *Candida* spp. Each infrared spectrum was compared with spectra stored in the software database. The updated version of the I-dOne software was used to analyze ATR-FTIR spectra. All *Candida* isolates 284/284 (100%) were classified correctly according to the genus. Overall species identification yielded 272/284 (95.8%) concordant identification results with MALDI-TOF MS. Additionally, all 79 isolates belonging to the *Candida parapsilosis* species complex were identified correctly to the species level with the updated version of the I-dOne software. Only 12 (4.2%) isolates were misidentified at the species level. The present study highlights the potential diagnostic performance of the I-dOne software with ATR-FTIR spectroscopic technique referral spectral database as a real alternative for routine identification of the most frequently isolated *Candida* spp.

## 1. Introduction

Over the past few decades, the epidemiology of fungal infections has substantially changed as the proportion of the population at risk has increased [1,2,3,4]. In fact, the prevalence and incidence of nosocomial and healthcare-associated fungal infections, mainly due to *Candida* spp. and *Aspergillus* spp., have witnessed an outstanding rise [1,2,3].

Invasive *Candida* spp. infections affect over 250,000 immunocompromised patients per year worldwide and are responsible for over 50,000 deaths [5,6]. Among healthcare-associated bloodstream infections, candidemia is the fourth most common cause [2,5,7]. The impact of such detrimental opportunistic infections bears dramatic consequences for both patients and the healthcare economy. Indeed, the mortality rate due to *Candida* infections ranged from 35% to 64% [5,6,8], and hospitalization cost was estimated to be around USD 1.4 billion in 2017 in the USA [9].

By looking at species distribution, *Candida albicans* remains the most frequently isolated fungal pathogen from blood cultures [10]. Non-*albicans Candida* species distribution differs greatly from one country to another [11,12]. According to recent reports, *Candida glabrata* was the second most frequently isolated *Candida* species from blood cultures in Canada and North America [13,14]. In particular, the prevalence of *C. glabrata* causing candidemia in the USA increased from 12% in 1992–1993 to 27% in the late 2000s [15]. Clinical surveys from several countries in Southern Europe reported *Candida parapsilosis* as the second most frequently isolated *Candida* species from blood cultures and in certain scenarios even the first [8,16,17,18,19]. Correlated to the increase in the prevalence and incidence of non-*albicans Candida* species among bloodstream infections, researchers have highlighted that drug resistance rates are also increasing over time as they represent the focus of current clinical research efforts [20,21,22,23].

First-line laboratory approach to differentiate *Candida* species could involve the use of chromogenic media. However, considering that the phenotypic characteristics of different *Candida* species might be similar, this phenotypic test can only help microbiologists in presumptive species identification, and it is therefore mandatory to confirm the first diagnostic hypothesis with a second molecular approach [24]. To this point, matrix-assisted laser desorption/ionization time-of-flight mass spectrometry (MALDI-TOF MS) has been extensively used in routine diagnosis [25]. MALDI-TOF MS can also be performed directly on blood cultures that tested positive for yeast after Gram staining [26], yielding results in a few minutes [27].

A new approach in microbial identification is Fourier transform infrared (FTIR) spectroscopy [28]. This is a spectrum-based technique, first introduced in the 1950s, that, within its wide spectrum of applications, can quantify the absorption of infrared light by molecules present in the microbial cell. The generated IR spectrum provides a specific fingerprint that reflects the cell composition of nucleic acids, proteins, lipids, and carbohydrates [28,29]. Each microorganism has a highly specific infrared absorption chemical signature correlated with genetic information, thus allowing for the identification of the microbial species [30]. The attenuated total reflection (ATR) technique is the most frequently used to investigate the chemical composition of intact microorganisms from a monomicrobial culture grown on solid media [29]. In the ATR method, the IR beam is directed toward an ATR crystal at a defined angle, whereby the total internal reflection occurs within the crystal. Under these conditions, an evanescent wave extends beyond the surface of the ATR crystal only a few microns (0.5–5 mm). The interaction of the evanescent wave with the intact microbial cells previously deposited on the ATR crystal results in the partial attenuation of the total internally reflected IR beam at the wavelengths at which the sample absorbs the IR energy [31]. The attenuated evanescent wave passes back to the IR beam, which exits the opposite end of the crystal to reach the detector in the IR spectrometer. The generated infrared spectrum enables microbial identification. Promising results, using ATR-FTIR spectroscopy, were reported for both bacterial and yeast identification and showed the potential for the discrimination of antibiotic- and antifungal-resistant strains [32,33,34,35].

The aim of the present study was to evaluate the performance of the I-dOne software (Alifax, Polverara (PD)—Italy) for the classification of ATR-FTIR spectra leading to the identification of clinical yeast isolates in comparison with MALDI-TOF MS, the method currently used in our laboratory.

Furthermore, a prototype version of the I-dOne software (Research Use Only, RUO) was used to evaluate the method’s capability to correctly classify the members of the *Candida parapsilosis* complex at the species level.

## 2. Materials and Methods

### 2.1. Study Design and Settings

This is a prospective methodological study conducted from January 2021 to December 2023, at Pisa University Hospital, a tertiary-level care center hosting over 1108 beds and nearly 50,000 admissions per year in 2021. Our clinical epidemiology regarding invasive fungal infections and species distribution has been described elsewhere [18,35]. Isolates were subcultured at 37 °C for 24 h on Sabouraud agar (Biomérieux, Craponne, France). The routine identification of isolated yeast colonies was performed by MALDI-TOF MS (Bruker Corporation, Billerica, MA, USA). In parallel, yeast grown on solid media were spotted onto the ATR crystal for identification by FTIR spectroscopy and interpretation by I-dOne software. All the 284 isolates included in this study were first analyzed with MALDI-TOF and then reassessed with ATR-FTIR using the I-dOne software.

### 2.2. Yeasts

Yeast isolates were collected at Pisa University Hospital, Mycology Unit, and from the yeast collection of the Department of Biology, University of Pisa, where isolates were kept frozen at −80 °C in brain heart broth with 10% glycerol. For ATR-FTIR spectroscopy, yeast colonies were cultured on solid Tryptic Soy Agar, Sabouraud–gentamycin–chloramphenicol agar, chromID CPSE agar, and Oxoid Chromogenic Candida Agar (Biomérieux) at 37 °C for 24–48 h depending on the species.

### 2.3. Sample Preparation for ATR-FTIR Spectroscopy

For ATR-FTIR spectroscopy, the spectral acquisition protocol involved background spectrum acquisition prior to the preparation of each sample, in order to compensate for the fluctuation of environmental humidity. Then, a single yeast colony was isolated using a sterile disposable loop and deposited directly onto the ATR crystal sampling surface of the ATR-FTIR spectrometer for the analysis. After sample deposition, a short time was necessary to permit the evaporation of moisture residues from the sample. The sample’s drying was automatically evaluated by the software, and this was necessary to avoid elevated IR absorption by water in the protein region of the infrared spectra. In addition, using the appropriate incorporated sample press, external extra pressure was applied for yeast colonies to ensure good contact between microbial cells and the ATR crystal. After the spectral acquisition, the ATR sampling surface was cleaned with 70% ethanol according to the manufacturer’s instructions in order to remove the organic components of the previously analyzed yeast. The variability in the IR analysis of microorganisms among the yeast spectra belonging to the same species had to be less than the one between the spectra of yeasts from different species. Spectral reproducibility depends mostly on rigorous control of sampling and measurement conditions: the composition of the growth medium and the phase of growth are crucial, as relative peak intensities may be more affected than peak positions, since metabolite production may change over different growth phases according to the available nutrients [36,37]. Spectral acquisition time was approximately 60 s, and the automated spectral processing and the identification of the isolate were completed in another 30 s. The identification process required from 1 to 3 replicates for each sample.

### 2.4. ATR-FTIR Spectral Acquisition

All spectra ranging between wave numbers 4000 and 400 cm^−1^ were recorded with an ATR-FTIR spectrometer (Agilent, Santa Clara, CA, USA). The spectral acquisition was reagent-free, and only isopropanol was required as reference spectra and ethanol 70% for the cleaning of the ATR crystal. For data processing, the I-dOne software (version 2.0) was used for the microbiological classification of yeast species. For the subclassification of the *Candida parapsilosis complex* into each separate species, a custom-developed RUO version of the I-dOne algorithm was used.

### 2.5. Data Processing

To compare the spectra of the different *Candida* species, spectra were classified by using a hierarchical classification algorithm, which is capable of classifying the unknown microorganism in progressive subgroups until the final species classification. During this cyclic classification process, all the microbial features (proteins, lipids, carbohydrates, and nucleic acids) are used to distinguish between different groups. The data analysis protocol is fully covered by patent [38]; hence, more in-depth details are not discussed.

## 3. Results

### Identification of Clinical Yeasts by ATR-FTIR Spectroscopy

All 284 isolates were identified at MALDI-TOF analyses, and identification was performed in technical duplicates. The total number of *Candida* species used in the present study was 284. Of these, 139 (48.9%) were *C. albicans*, 66 (23.2%) were *Candida parapsilosis* species complex (47 *Candida parapsilosis*, 10 *Candida metapsilosis*, and 9 *Candida orthopsilosis*); 50 (17.6%) were *Candida glabrata*, 20 (7%) were *Candida tropicalis*, and 9 (3.2%) were *Candida krusei*. The ATR-FTIR spectral reference database initially contained the spectra of isolates belonging to the five most relevant *Candida* species: *C. albicans*, *C. glabrata*, *C. parapsilosis* species complex, *C. tropicalis*, and *C. krusei*. Next, an updated Research Use Only version of the I-dOne software was used to perform spectra analysis on the *Candida parapsilosis* species complex.

Identification performed by I-dOne software with the ATR-FTIR spectral reference database yielded 284 (100%) out of 284 concordant genus identification results. On a species level, 272 (95.8%) out of the 284 isolates showed concordant identification results with those obtained by MALDI-TOF MS. Identification results by the I-dOne software performed on the ATR-FTIR spectral reference database revealed that 2 (0.7%) out of the 284 yeast isolates were reported as “Candida species”. All these (100%) were identified by MALDI-TOF MS as *Candida albicans*. In addition, 12 (4.2%) out of 284 isolates were not concordantly identified at the species level by the I-dOne software in comparison to MALDI-TOF (Table 1).

Concordance rates between the I-dOne software and MALDI-ToF MS analysis for the most frequently isolated *Candida* species were the following: 132 (95%) out of 139 *C. albicans*, 49 (98%) out of 50 *C. glabrata*, 47 (100%) out of 47 *C. parapsilosis*, 9 (100%) out of 9 *C. orthopsilosis*, 10 (100%) out of 10 *C. metapsilosis*, 9 (100%) out of 9 *C. krusei*, and 16 (80%) out of 20 *C. tropicalis*.

In 12 (4.2%) out of the 284 isolates, species were not concordantly identified between the I-dOne software and MALDI-TOF MS. Non-concordant identification results were the following: five *C. albicans* were identified as three *C. parapsilosis* species complex and two *C. glabrata*; one *C. glabrata* was identified as *C. krusei*; and four *C. tropicalis* were identified as two *C. albicans* and two *C. glabrata*. Data regarding not concordantly identified *Candida* isolates are shown in Table 1.

## 4. Discussion

The present study settled and then evaluated the diagnostic performance of the I-dOne software (Alifax, Polverara (PD)—Italy) with the ATR-FTIR spectroscopic technique regarding the identification of the most frequent yeast isolates encountered in clinical microbiology, in comparison to MALDI-TOF MS (Bruker Corporation, Billerica, MA, USA), which is the currently used method in our laboratory.

All the analyzed yeast isolates were concordantly identified by MALDI-TOF at the genus level, and 92.2% were identified at the species level. The time required for the analysis of each isolate was 90 s. Twelve isolates were not concordantly identified at the species level. Implementations done in the software upgrade and in the referral spectral library allowed for further species identification and discrimination within the C. parapsilosis species complex. This is a drawback of this molecular diagnostic technique hindering its clinical application up to this point. However, with these promising results, it is reasonable to believe that past issues with species identification within the *C. parapsilosis* species complex could be overcome as the I-dOne RUO software (Alifax) with the ATR-FTIR spectroscopic technique referral spectral database was able to concordantly identify all *C. parapsilosis* complex isolates: 100% *C. parapsilosis*, 100% *C. metapsilosis*, and 100% *C. orthopsilosis*.

Our results should, however, be interpreted with caution. The monocentric nature of the study and the research focus limited on the most commonly isolated yeast species in clinical practice available are all study limitations. In addition, it would be of the utmost importance to expand the analysis to *Candida auris*; therefore, the aim of future studies will be to pursue this goal. Conversely, the prospective nature of the study and the amplitude of the number of isolates analyzed, as well as the software implementation and upgrade delivering new insights on species identification within the *C. parapsilosis* species complex, are all points of strength.

Surprisingly, despite the relatively low rate of non-concordant species identification (3.9%), when we compared our results with major studies such as those reported by Lam et al. [36], our overall species non-concordant identification rate was higher (3.9% vs. 0%). In previous studies reported by the same authors, the non-concordant identification rate was still lower (0.9%) than the one reported in our study [31]. However, both aforementioned studies used an optimized algorithm, specifically for Sabouraud agar.

Based on the results presented in this study, it can be stated that the I-dOne software (Alifax) with the ATR-FTIR spectroscopic technique referral spectral database could be a reliable alternative to MALDI-TOF MS analysis for the molecular identification of *Candida* spp. in a clinical laboratory routine workflow. Nevertheless, these results should be further investigated with other studies comparing ATR-FTIR with current clinical reference standards on yeast identification and speciation from other working groups.

## 5. Conclusions

The I-dOne software with ATR-FTIR was found to be a potential real alternative for routine molecular biology and biochemical analysis for the rapid microbial identification of yeast isolates in a clinical laboratory, as it is fast and easy to perform. Nevertheless, confirmatory studies and investigation regarding diagnostic performance on less frequently isolated yeast species should be the aim of future studies.

## Figures and Tables

**Table 1:** Identification of 284 *Candida* spp. isolates by I-dOne software with ATR-FTIR spectroscopy.

*Candida* Species	Concordant Results *		Non-Concordant Identification at the Species Level		Concordant Identification at the Genus Level		Total Isolates	
	N	%	N	%	N	%	N	%
*Candida albicans*	132	95.0%	5 ^a^	3.6%	2	1.4%	139	100.0%
*Candida glabrata*	49	98.0%	1 ^b^	2.0%		0.0%	50	100.0%
*Candida krusei*	9	100.0%		0.0%		0.0%	9	100.0%
*Candida metapsilosis*	10	100.0%		0.0%		0.0%	10	100.0%
*Candida orthopsilosis*	9	100.0%		0.0%		0.0%	9	100.0%
*Candida parapsilosis*	47	100.0%		0.0%		0.0%	47	100.0%
*Candida tropicalis*	16	80.0%	4 ^c^	20.0%		0.0%	20	100.0%
Overall	272	95.8%	10	3.5%	2	0.7%	284	100.0%

* Using the results from the currently used method (MALDI-TOF) in our laboratory as reference. Species identified as follows: ^a^. 2 *C. glabrata*; 3 *C. parapsilosis complex*; ^b^. *C. krusei*; ^c^. 2 *C. albicans*; 2 *C. glabrata*.

## Data Availability

All necessary data required to evaluate the conclusions of this study are reported in the tables in the main text.

## References

[1] Seagle E.E., Williams S.L., Chiller T.M. (2021). Recent Trends in the Epidemiology of Fungal Infections. Infect. Dis. Clin. North Am..

[2] Suleyman G., Alangaden G.J. (2021). Nosocomial Fungal Infections: Epidemiology, Infection Control, and Prevention. Infect. Dis. Clin. North Am..

[3] Bongomin F., Gago S., Oladele R.O., Denning D.W. (2017). Global and Multi-National Prevalence of Fungal Diseases-Estimate Precision. J. Fungi.

[4] Casadevall A. (2018). Fungal Diseases in the 21st Century: The Near and Far Horizons. Pathog. Immun..

[5] Pappas P.G., Lionakis M.S., Arendrup M.C., Ostrosky-Zeichner L., Kullberg B.J. (2018). Invasive Candidiasis. Nat. Rev. Dis. Primers.

[6] Kullberg B.J., Arendrup M.C. (2015). Invasive Candidiasis. N. Engl. J. Med..

[7] Wisplinghoff H., Bischoff T., Tallent S.M., Seifert H., Wenzel R.P., Edmond M.B. (2004). Nosocomial Bloodstream Infections in US Hospitals: Analysis of 24,179 Cases from a Prospective Nationwide Surveillance Study. Clin. Infect. Dis..

[8] Mete B., Zerdali E.Y., Aygun G., Saltoglu N., Balkan I.I., Karaali R., Kaya S.Y., Karaismailoglu B., Kaya A., Urkmez S. (2021). Change in Species Distribution and Antifungal Susceptibility of Candidemias in an Intensive Care Unit of a University Hospital (10-Year Experience). Eur. J. Clin. Microbiol. Infect. Dis..

[9] Benedict K., Jackson B.R., Chiller T., Beer K.D. (2019). Estimation of Direct Healthcare Costs of Fungal Diseases in the United States. Clin. Infect. Dis..

[10] Pfaller M.A., Diekema D.J., Turnidge J.D., Castanheira M., Jones R.N. (2019). Twenty Years of the SENTRY Antifungal Surveillance Program: Results for *Candida* Species From 1997–2016. Open Forum Infect Dis.

[11] Toda M. (2019). Population-Based Active Surveillance for Culture-Confirmed Candidemia—Four Sites, United States, 2012–2016. MMWR Surveill Summ.

[12] Lamoth F., Lockhart S.R., Berkow E.L., Calandra T. (2018). Changes in the Epidemiological Landscape of Invasive Candidiasis. J. Antimicrob. Chemother..

[13] Guinea J. (2014). Global Trends in the Distribution of *Candida* Species Causing Candidemia. Clin. Microbiol. Infect..

[14] Vallabhaneni S., Cleveland A.A., Farley M.M., Harrison L.H., Schaffner W., Beldavs Z.G., Derado G., Pham C.D., Lockhart S.R., Smith R.M. (2015). Epidemiology and Risk Factors for Echinocandin Nonsusceptible *Candida glabrata* Bloodstream Infections: Data from a Large Multisite Population-Based Candidemia Surveillance Program, 2008–2014. Open Forum Infect. Dis..

[15] Lockhart S.R., Iqbal N., Cleveland A.A., Farley M.M., Harrison L.H., Bolden C.B., Baughman W., Stein B., Hollick R., Park B.J. (2012). Species Identification and Antifungal Susceptibility Testing of *Candida* Bloodstream Isolates from Population-Based Surveillance Studies in Two U.S. Cities from 2008 to 2011. J. Clin. Microbiol..

[16] Mesini A., Mikulska M., Giacobbe D.R., Del Puente F., Gandolfo N., Codda G., Orsi A., Tassinari F., Beltramini S., Marchese A. (2020). Changing Epidemiology of Candidaemia: Increase in Fluconazole-Resistant *Candida parapsilosis*. Mycoses.

[17] Siopi M., Tarpatzi A., Kalogeropoulou E., Damianidou S., Vasilakopoulou A., Vourli S., Pournaras S., Meletiadis J. (2020). Epidemiological Trends of Fungemia in Greece with a Focus on Candidemia during the Recent Financial Crisis: A 10-Year Survey in a Tertiary Care Academic Hospital and Review of Literature. Antimicrob. Agents Chemother..

[18] Franconi I., Rizzato C., Tavanti A., Falcone M., Lupetti A. (2023). Paradigm Shift: *Candida parapsilosis* Sensu Stricto as the Most Prevalent *Candida* Species Isolated from Bloodstream Infections with Increasing Azole-Non-Susceptibility Rates: Trends from 2015-2022 Survey. J. Fungi.

[19] Govender N.P., Patel J., Magobo R.E., Naicker S., Wadula J., Whitelaw A., Coovadia Y., Kularatne R., Govind C., Lockhart S.R. (2016). Emergence of Azole-Resistant *Candida parapsilosis* Causing Bloodstream Infection: Results from Laboratory-Based Sentinel Surveillance in South Africa. J. Antimicrob. Chemother..

[20] Perlin D.S., Rautemaa-Richardson R., Alastruey-Izquierdo A. (2017). The Global Problem of Antifungal Resistance: Prevalence, Mechanisms, and Management. Lancet Infect. Dis..

[21] Franconi I., Rizzato C., Poma N., Tavanti A., Lupetti A. (2023). *Candida parapsilosis* Sensu Stricto Antifungal Resistance Mechanisms and Associated Epidemiology. J. Fungi.

[22] Franconi I., Lupetti A. (2023). In Vitro Susceptibility Tests in the Context of Antifungal Resistance: Beyond Minimum Inhibitory Concentration in *Candida* Spp. J. Fungi.

[23] Daneshnia F., de Almeida Júnior J.N., Ilkit M., Lombardi L., Perry A.M., Gao M., Nobile C.J., Egger M., Perlin D.S., Zhai B. (2023). Worldwide Emergence of Fluconazole-Resistant *Candida parapsilosis*: Current Framework and Future Research Roadmap. Lancet Microbe.

[24] Leena Sankari S., Mahalakshmi K., Naveen Kumar V. (2019). Chromogenic Medium versus PCR-RFLP in the Speciation of *Candida*: A Comparative Study. BMC Res. Notes.

[25] Patel R. (2019). A Moldy Application of MALDI: MALDI-ToF Mass Spectrometry for Fungal Identification. J. Fungi.

[26] Vecchione A., Florio W., Celandroni F., Barnini S., Lupetti A., Ghelardi E. (2018). A Rapid Procedure for Identification and Antifungal Susceptibility Testing of Yeasts from Positive Blood Cultures. Front. Microbiol..

[27] Clark A.E., Kaleta E.J., Arora A., Wolk D.M. (2013). Matrix-Assisted Laser Desorption Ionization–Time of Flight Mass Spectrometry: A Fundamental Shift in the Routine Practice of Clinical Microbiology. Clin. Microbiol. Rev..

[28] Quintelas C., Ferreira E.C., Lopes J.A., Sousa C. (2018). An Overview of the Evolution of Infrared Spectroscopy Applied to Bacterial Typing. Biotechnol. J..

[29] Rakovitsky N., Frenk S., Kon H., Schwartz D., Temkin E., Solter E., Paikin S., Cohen R., Schwaber M.J., Carmeli Y. (2020). Fourier Transform Infrared Spectroscopy Is a New Option for Outbreak Investigation: A Retrospective Analysis of an Extended-Spectrum-Beta-Lactamase-Producing Klebsiella Pneumoniae Outbreak in a Neonatal Intensive Care Unit. J. Clin. Microbiol..

[30] Wenning M., Scherer S. (2013). Identification of Microorganisms by FTIR Spectroscopy: Perspectives and Limitations of the Method. Appl. Microbiol. Biotechnol..

[31] Lam L.M.T., Dufresne P.J., Longtin J., Sedman J., Ismail A.A. (2019). Reagent-Free Identification of Clinical Yeasts by Use of Attenuated Total Reflectance Fourier Transform Infrared Spectroscopy. J. Clin. Microbiol..

[32] Amiali N.M., Mulvey M.R., Berger-Bächi B., Sedman J., Simor A.E., Ismail A.A. (2008). Evaluation of Fourier Transform Infrared Spectroscopy for the Rapid Identification of Glycopeptide-Intermediate Staphylococcus Aureus. J. Antimicrob. Chemother..

[33] Bosch A., Miñán A., Vescina C., Degrossi J., Gatti B., Montanaro P., Messina M., Franco M., Vay C., Schmitt J. (2008). Fourier Transform Infrared Spectroscopy for Rapid Identification of Nonfermenting Gram-Negative Bacteria Isolated from Sputum Samples from Cystic Fibrosis Patients. J. Clin. Microbiol..

[34] Sandt C., Madoulet C., Kohler A., Allouch P., De Champs C., Manfait M., Sockalingum G.D. (2006). FT-IR Microspectroscopy for Early Identification of Some Clinically Relevant Pathogens. J. Appl. Microbiol..

[35] De Carolis E., Magrì C., Camarlinghi G., Ivagnes V., Spruijtenburg B., Meijer E.F.J., Scarselli C., Parisio E.M., Sanguinetti M. (2024). Follow the Path: Unveiling an Azole Resistant *Candida parapsilosis* Outbreak by FTIR Spectroscopy and STR Analysis. J. Fungi.

[36] Lam L.M.T., Ismail A.A., Lévesque S., Dufresne S.F., Cheng M.P., Vallières É., Luong M.-L., Sedman J., Dufresne P.J. (2022). Multicenter Evaluation of Attenuated Total Reflectance Fourier Transform Infrared (ATR-FTIR) Spectroscopy-Based Method for Rapid Identification of Clinically Relevant Yeasts. J. Clin. Microbiol..

[37] Baldauf N.A., Rodriguez-Romo L.A., Männig A., Yousef A.E., Rodriguez-Saona L.E. (2007). Effect of Selective Growth Media on the Differentiation of Salmonella Enterica Serovars by Fourier-Transform Mid-Infrared Spectroscopy. J. Microbiol. Methods.

[38] (2020). Alifax, S. r. l. I-DOne: User’s Manual. https://www.alifax.net/prodotti/batteriologica/show/I-done.

